# Supplementary material for: Prefrontal–bed nucleus of the stria terminalis physiological and neuropsychological biomarkers predict therapeutic outcomes in depression
Source: Nat Commun. 2025 Nov 18;16:10034. doi: 10.1038/s41467-025-65179-z (PMC12627847; doi:10.1038/s41467-025-65179-z)
Supplement: Supplementary file 1 — Supplementary Information [file 41467_2025_65179_MOESM1_ESM.pdf]

Supplementary Information for

Prefrontal–Bed Nucleus of the Stria Terminalis Physiological and Neuropsychological Biomarkers Predict Therapeutic Outcomes in Depression

Linbin Wang[1,2,3], Yingying Zhang[2], Yuhan Wang[1], Qiong Ding[3], Luling Dai[1], Kejia Hu[1], Kuanghao Ye[1], Xin Iv[1], Xiaoxiao Zhang[1], Alekhya Mandali[3], Luis Manssuer[3], Saurabh Sonkusare[3], Yijie Zhao[2], Peng Huang[1], Xian Qiu[1], Yixin Pan[1], Yijie Lai[1], Dianyou Li[1], Wei Liu[1], Shikun Zhan[1], Bomin Sun[1], Valerie Voon[1,2,3]

1 Department of Neurosurgery, Center for Functional Neurosurgery, Ruijin Hospital, Shanghai Jiao Tong University School of Medicine, Shanghai, China

2 Institute of Science and Technology for Brain-Inspired Intelligence (ISTBI), Fudan University, Shanghai, China

3 Department of Psychiatry, University of Cambridge, Cambridge, United Kingdom

Supplementary Table 1-4

Supplementary Figure 1-5

Supplementary Table 1: Demographic and clinical characteristics

Partients	Education (y)	Age at surgery (y)	Occupation state	Gender	Disease duration (y)	No. of previous episodes	No. of antidepressant drugs at surgery	No. of previous hospitalizations	MoCA	HAMD
**MDDDBS01†**	15	31	unemployed	Male	11	1	2	2	30	19
**MDDDBS02†,#,***	11	37	employed	Male	19	1	4	2	27	17
**MDDDBS03†,#,***	9	49	employed	Male	33	1	1	4	27	23
**MDDDBS04†,#**	17	35	employed	Male	18	1	5	1	26	23
**MDDDBS05†,#**	11	45	employed	Male	23	1	3	6	26	19
**MDDDBS06**	7	34	employed	Male	14	2	2	2	27	17
**MDDDBS07**	13	19	student (schooling suspension)	Female	4	1	5	1	22	22
**MDDDBS08†,#,***	13	34	unemployed	Male	19	1	1	9	28	20
**MDDDBS09**	14	24	student (schooling suspensio	Male	4	1	6	3	27	19

			n)							
**MDDD BS10[†,#]**	13	30	unemployed	Male	15	1	2	1	26	26
**MDDD BS11**	9	38	unemployed	Male	13	1	0	3	24	20
**MDDD BS12[†,#,*]**	17	57	employed	Male	25	3	2	3	24	18
**MDDD BS13[†,#,*]**	16	37	employed	Male	18	4	2	7	29	22
**MDDD BS14**	15	24	unemployed	Male	10	1		3	28	23
**MDDD BS15[†,#]**	9	18	unemployed	Female	5	1	1	2	28	19
**MDDD BS16[†,#]**	11	32	unemployed	Male	17	1	3	3	26	23
**MDDD BS17**	12	16	student (schooling suspension)	Male	1	1	4	3	30	27
**MDDD BS18[†,#,*]**	12	32	employed	Male	14	1	1	2	26	19
**MDDD BS19[†]**	9	20	unemployed	Female	10	1	3	3	30	28
**MDDD BS20[†,#]**	15	41	employed	Male	28	1	0[a]	3	24	19
**MDDD BS21[†,#,*]**	9	31	unemployed	Male	8	1	2	2	22	29
**MDDD BS22[†,#]**	20	35	unemployed	Male	19	1	0[a]	2	27	28

**MDDDBS23†, #**	16	29	employed	Male	5	2	3	3	30	19
**MDDDBS24***	14	21	student	Female	7	1	3	1	24	17
**MDDDBS25***	14	23	unemployed	Male	7	2	1	3	26	17
**MDDDBS26**	12	24	unemployed	Male	7	1	3	2	26	20

†Eyes-closed dataset, #Eyes-open dataset, *Wireless dataset, [a]Not effective, refused to take.

Supplementary Table 2: BNST-NAc DBS induced volume of tissue activated (VTA) and its overlap with (left and right) BNST and NAc at the last follow-up

Patients	right BNST	left BNST	right NAc	left NAc
MDDDBS01	18.1728379	17.9233419	0.08978675	4.50634307
MDDDBS02	3.9079415	16.9013583	0	2.07207869
MDDDBS03	0	2.92126056	0	0
MDDDBS04	7.82041425	12.0836152	0.20084531	0
MDDDBS05	0.41227087	12.1303645	0	0
MDDDBS06	3.27888617	3.87947423	0	0
MDDDBS07	1.76308521	7.95837131	10.8135893	32.6735355
MDDDBS08	1.79246996	11.473886	0	13.5779797
MDDDBS09	7.13866575	6.03289039	0	0
MDDDBS10	5.55660433	10.0275457	0	1.99265332
MDDDBS11	17.4271104	18.3535019	0.04849845	4.57150646
MDDDBS12	2.61984079	2.50182318	0	0
MDDDBS13	0	2.98345244	0	0
MDDDBS14	0.60311316	15.8634019	0	0
MDDDBS15	0.59676452	8.7597756	0	0
MDDDBS16	1.00230134	18.3824787	0	1.75152202
MDDDBS17	26.1333873	3.43801708	0.69380674	2.9865603
MDDDBS18	7.20305134	6.59504154	0	0.86601556
MDDDBS19	2.13818632	8.18026172	0	0
MDDDBS20	14.0801973	10.1989579	0	0.37139062
MDDDBS21	2.04208616	4.76028568	0.07484085	1.37854427
MDDDBS22	7.07298309	63.3659962	0	0.44004164
MDDDBS23	11.7359446	12.026042	2.41403923	1.52166246
MDDDBS24	12.9695846	19.6454431	5.69396398	3.88947361
MDDDBS25	4.82154786	14.9541858	0	0.04142434
MDDDBS26	0.88154269	4.32683112	0	0

Supplementary Table 3: Stimulation parameters

Patients	Stimulation Parameters
MDDDBS01	L: C+4-5-6-7-/5.7V/210µs/160HzIR: C+12-13-14-/5.7V/210µs/160Hz
MDDDBS02	L: C+11-12-13-/5.5V/210µs/180HzIR: C+3-4-5-/5.5V/210µs/180Hz
MDDDBS03	L: C+13-14-/4.25V/210µs/160HzIR: C+5-6-/4.25V/210µs/160Hz
MDDDBS04	L: C+12-14-/4.8V/180µs/150HzI R: C+4-6-/4.8V/180µs/150Hz (07:00-23:00)<br>L: C+11-12-/3.5V/150µs/160HzI R: C+3-4-/3.5V/150µs/160Hz (23:00-07:00)
MDDDBS05	L: C+5-7-/3.8V/120µs/180HzI R: C+13-15-/3.8V/120µs/180Hz
MDDDBS06	L: C+3-5-7-/5.5V/210µs/160HzI R: C+11-13-15-/5.5V/210µs/160Hz
MDDDBS07	L: C+3-4-5-/5.5V/150µs/160HzI R: C+11-12-13-/5.5V/150µs/160Hz
MDDDBS08	L: C+10-11-13-14-/5.45V/190µs/180HzI R: C+3-4-/5.45V/190µs/180Hz (06:30-22:00)<br>L: C+10-11-13-14-/3.50V/180µs/180HzI R: C+3-4-/3.5V/180µs/180Hz (22:00-06:30)
MDDDBS09	L: C+13-15-/3.8V/210µs/160HzI R: C+5-7-/3.8V/210µs/160Hz
MDDDBS10	L: C+2-3-4-/4.7V/210µs/180HzI R: C+10-11-12-/4.7V/210µs/175Hz
MDDDBS11	L: C+11-12-13-/5.3V/210µs/160HzI R: C+3-4-5-/5.3V/210µs/160Hz (06:00-12:00)<br>L: C+11-12-14-/5.6V/210µs/180HzI R: C+3-4-6-/5.6V/210µs/180Hz (12:00-20:00)<br>L: C+12-13-14-/4.0V/150µs/160HzI R: C+4-5-6-/4.0V/150µs/160Hz (20:00-06:00)
MDDDBS12	L: C+14-15-/4.8V/180µs/160HzI R: C+6-7-/4.8V/180µs/160Hz
MDDDBS13	L: C+14-15-/3.5V/180µs/160HzI R: C+6-7-/3.5V/180µs/160Hz
MDDDBS14	L: C+11-13-15-/4.25V/180µs/160HzI R: C+2-4-/4.25V/180µs/160Hz
MDDDBS15	L:C+11-12-13-/4.2V/160µs/160HzI R: C+3-4-6-/4.2V/160µs/160Hz (07:00-24:00)<br>L: C+11-13-/2.5V/150µs/160HzI R: C+4-6-/2.5V/150µs/160Hz (00:00-07:00)
MDDDBS16	L:C+12-14-15-/5.5V/150µs/180HzI R: C+4-6-7-/5.5V/150µs/180Hz (09:00-22:00)<br>L: C+12-15-/3.8V/150µs/180HzI R: C+3-6-/3.8V/150µs/180Hz (22:00-09:00)

MDDDBS17	L: C+11-12-/4.0V/150µs/160Hzl R: C+3-4-/4.70V/150µs/160Hz (07:00-21:00)<br>L: C+14-15-/3.0V/150µs/160Hzl R: C+6-7-/3.0V/150µs/160Hz (21:00-07:00)
MDDDBS18	L: C+12-/4.7V/150µs/160Hzl R: C+4-/4.7V/150µs/160Hz (07:30-22:00)<br>L: C+13-/3.5V/150µs/160Hzl R: C+5-/3.5V/150µs/160Hz (22:00-07:30)
MDDDBS19	L: C+11-12-/4.0V/150µs/160Hzl R: C+3-4-/4.0V/150µs/160Hz (09:30-24:00)<br>L: C+11-/3.0V/180µs/160Hzl R: C+2-4-/3.0V/180µs/160Hz (00:00-09:30)
MDDDBS20	L: C+12-14-/4.5V/150µs/160Hzl R: C+4-6-/4.55V/150µs/160Hz
MDDDBS21	L: C+12-14-/4.8V/210µs/170Hzl R: C+4-6-/4.8V/210µs/170Hz (07:30-24:00)<br>L: C+12-14-/4.0V/180µs/160Hzl R: C+4-6-/4.0V/180µs/160Hz (00:00-07:30)
MDDDBS22	L: C+11-13-/4.0V/180µs/160Hzl R: C+3-5-/4.0V/180µs/160Hz
MDDDBS23	L: C+10-11-/5.7V/150µs/180Hzl R: C+2-3-4-/5.7V/150µs/180Hz
MDDDBS24	L: C+10-11-12-13-/5.6V/210µs/180Hz lR: C+2-3-4-5-/5.9V/210µs/180Hz (08:30-03:00)<br>L: C+12-14-/4.0V/150µs/180Hzl R: C+4-6-/4.0V/150µs/180Hz (03:00-08:30)
MDDDBS25	L: C+3-5-/4.7V/210µs/170Hzl R: C+11-13-/3.5V/210µs/170Hz
MDDDBS26	L: C+11-13-/4.5V/180µs/180Hzl R: C+3-5-/4.5V/180µs/180Hz (07:00-23:00)<br>L: C+11-/2.0V/150µs/180Hzl R: C+3-/2.0V/150µs/180Hz (23:00-07:00)

Supplementary Table 4: Changes in the outcome measures during the open-label phase (n = 26)

Variable	Baseline Mean (SD)	LFU/T1 Mean (SD)	Improvement Mean (SD)	Changes Mean (SD)	95% CI	t	P value*
HAMD	21.3 (3.7)	11.2 (6.8)	47.2% (31.0%)	10.1 (7.1)	7.2~13.0	7.26	<0.0001
MADRS	31.5 (6.0)	18.0 (9.3)	44.2% (25.5%)	13.5 (7.8)	10.4~16.6	8.86	<0.0001
HAMA	24.0 (6.3)	13.6 (6.8)	43.6% (26.8%)	10.4 (7.2)	7.5~13.3	7.33	<0.0001
DARS	25.5 (12.0)	32.7 (14.7)	(-)46.6% (77.9%)	(-)7.2 (14.7)[a]	1.2~13.1	-2.48	0.0204
WHOQOL-BREF	30.6 (13.8)	42.2 (17.4)	(-)55.7% (82.3%)	(-)11.5 (14.2)[a]	5.8~17.3	-4.15	0.0003
SF-36	266.7 (107.6)	383.1 (177.5)	(-)56.3% (70.9%)	(-)116.4 (162.7)[a]	50.6~182.1	-3.65	0.0012
SDS	24.8 (5.8)	16.2 (9.0)	34.6% (40.9%)	8.6 (9.1)	4.9~12.3	4.8	0.0006

[a]Higher scores in DARS, WHOQOL-BREF and SF-36 means better state.

*Unadjusted p value for paired student t-tests (two-sided).

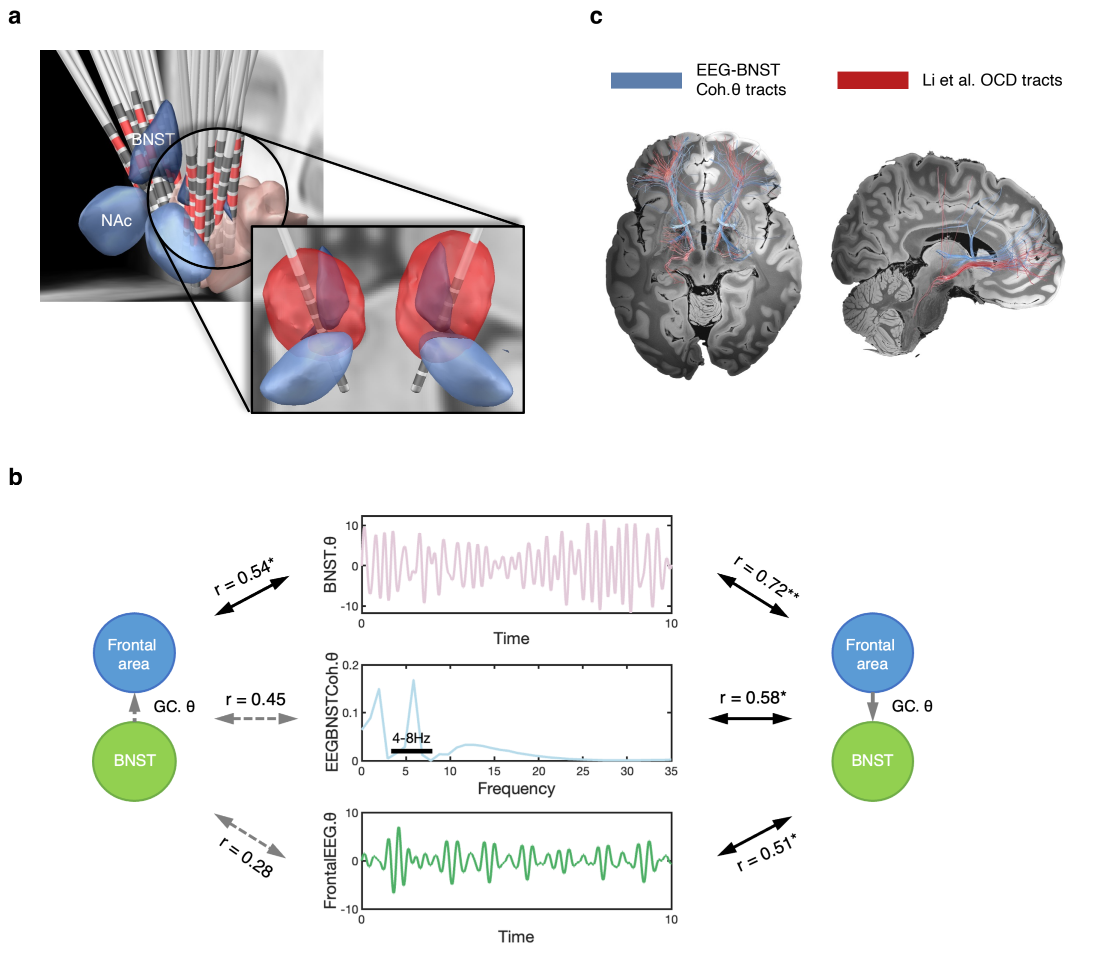

Supplementary Fig. 1: Frontal–BNST theta connectivity. a. Intracranial electrodes (n = 26) with monopolar stimulation sites (red) and example BNST-NAc DBS induced VTA at final follow-up. **b.** Granger causality (GC) of theta band of top-down (Frontal EEG -> BNST) and bottom-up (BNST -> Frontal EEG) and correlations with frontal EEG and BNST theta power and frontal-BNST theta coherence. Unadjusted *$p < 0.05$, **$p < 0.01$ for Spearman's correlation (two-sided). **c.** Fiber tracts (blue) related to lower frontal EEG-BNST theta coherence power (predicting better clinical outcomes) overlaid with fiber tracts with the open-source tracts available for optimal OCD DBS targets (red). Abbreviations: Coh, coherence; GC; Granger Causality; OCD, obsessive–

compulsive disorder; BNST, bed nucleus of the stria terminalis, NAc, nucleus accumbens.

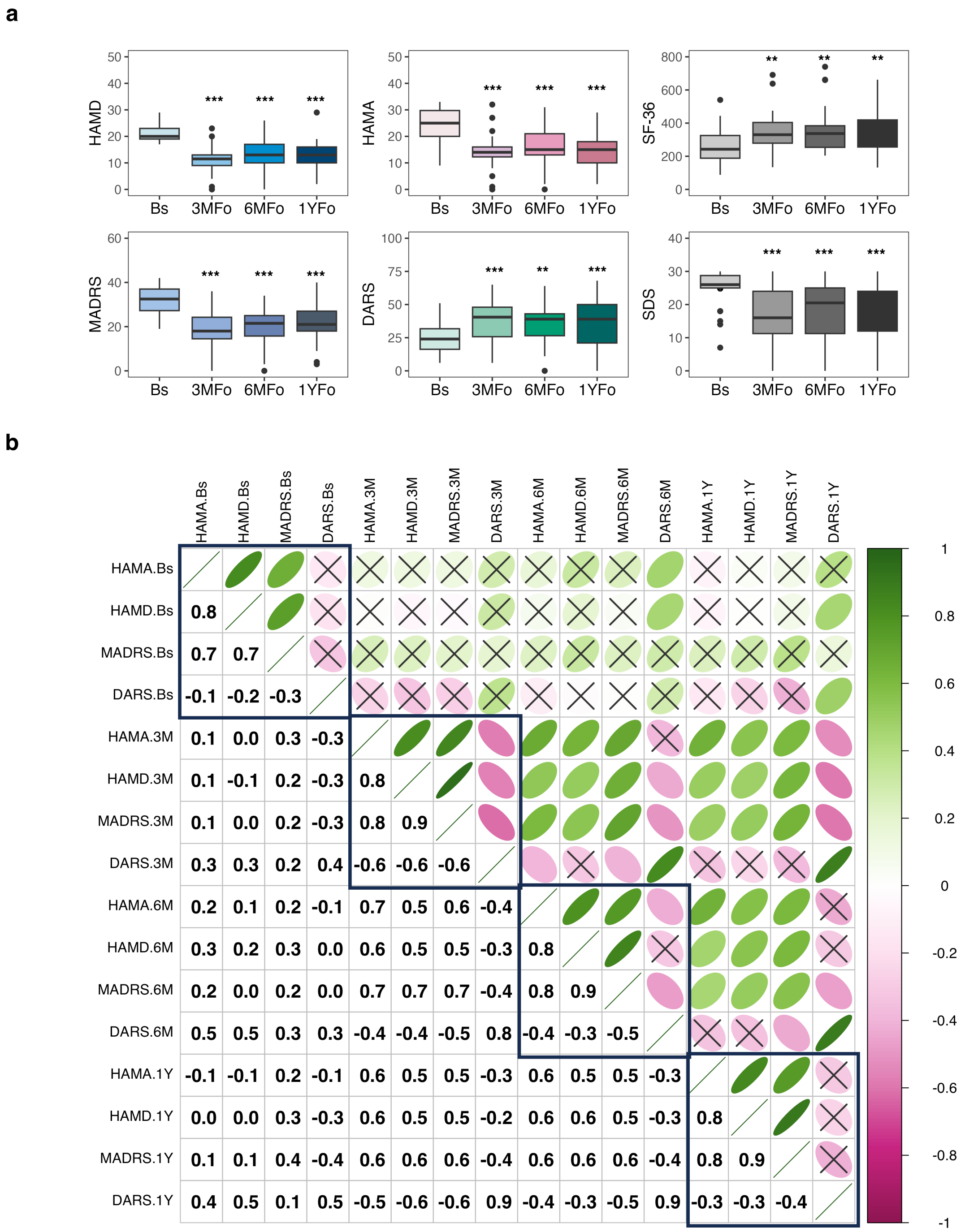

Supplementary Fig. 2: Clinical outcomes. a. Depression (HAMD, MADRS), anxiety (HAMA), anhedonia (DARS), quality-of-life (SF-36) and disability (SDS) scores at pre- versus postoperative 3-month (n = 26), 6-month (n = 26) and 1-year (n = 21). Unadjusted *p < 0.05, **p < 0.01, ***p < 0.001 for paired student t test (two-sided). **b.** Spearman's correlation matrix of the depression (HAMD,

MADRS), anxiety (HAMA) and anhedonia (DARS) scores at pre- and postoperative 3-month (n = 26), 6-month (n = 26) and 1-year (n = 21). X symbol indicates non-significant (two-sided). Abbreviations: HAMD, Hamilton Depression Scale; HAMA, Hamilton Anxiety Scale; MADRS, Montgomery-Asberg Depression Rating Scale; DARS, Dimensional Anhedonia Rating Scale; 3M, 3 months; 6M, 6 months; 12M, 12 months; fo, follow-up, bs, baseline.

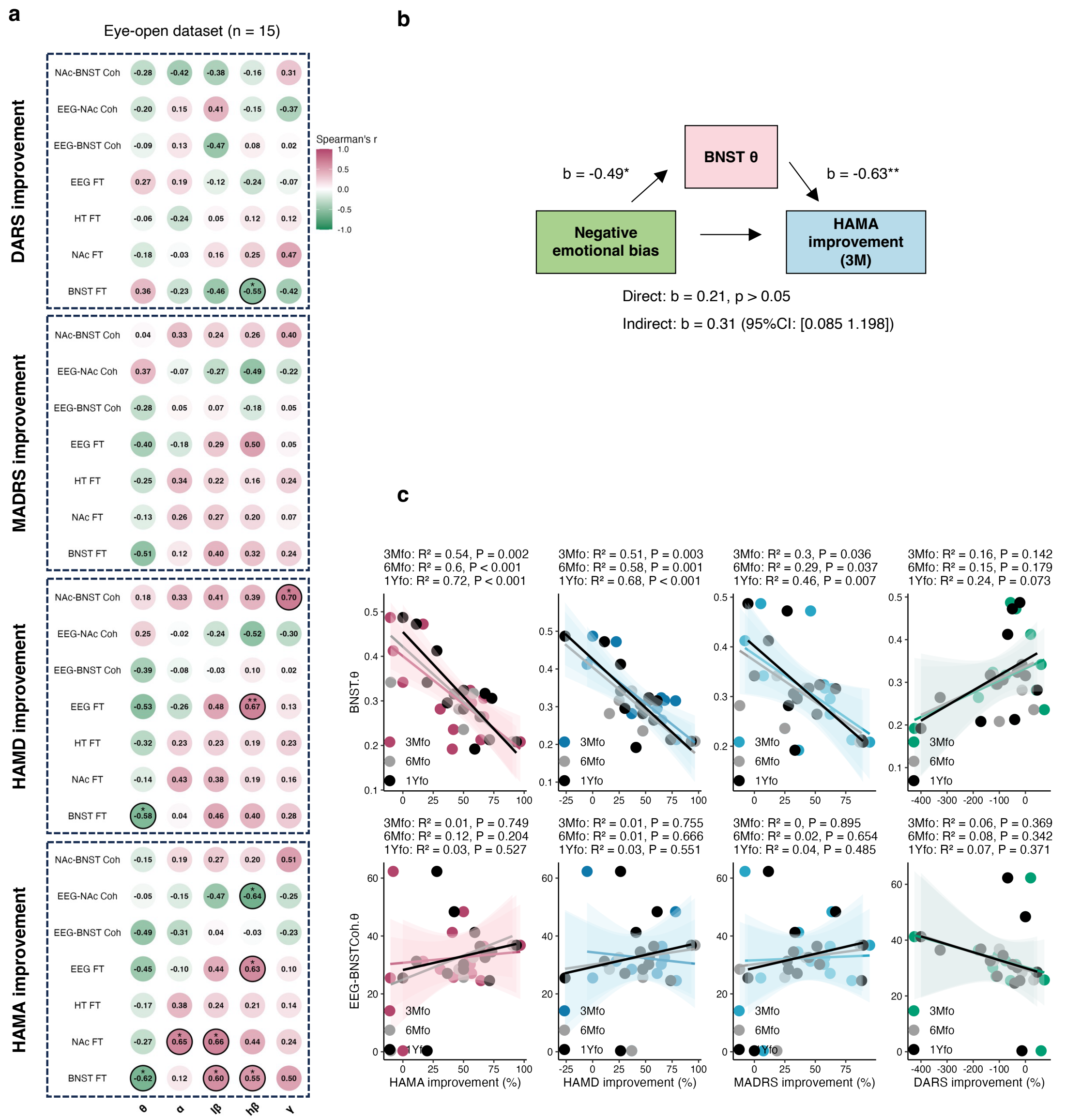

Supplementary Fig. 3: Biomarker validation in the eye open dataset. a. Correlation matrix between 3-month clinical improvements and spectral activity features in the eyes-open dataset (n = 15), black circle indicates unadjusted p < 0.05 for Spearman's correlation (two-sided). Source data are provided as a Source Data file. **b.** Mediation model of the indirect effect of negative emotional bias on clinical outcomes via theta biomarkers. *p < 0.05, **p < 0.01 for mediation test. **c.** Linear regression prediction of 3-month (n = 15), 6-month (n

= 15), 1-year (n = 14) clinical improvements by BNST theta power and theta coherence between frontal EEG and BNST in the eyes-open dataset. Regression line shows the fitted mean and shading represents 95% CI. Annotations report R^2 and P value for linear regression (two-sided). Abbreviations: HAMD, Hamilton Depression Scale; HAMA, Hamilton Anxiety Scale; MADRS, Montgomery-Asberg Depression Rating Scale; DARS, Dimensional Anhedonia Rating Scale; 3M, 3 months; 6M, 6 months; 12M, 12 months; fo, follow-up; FT, Fourier Transform; Coh, coherence.

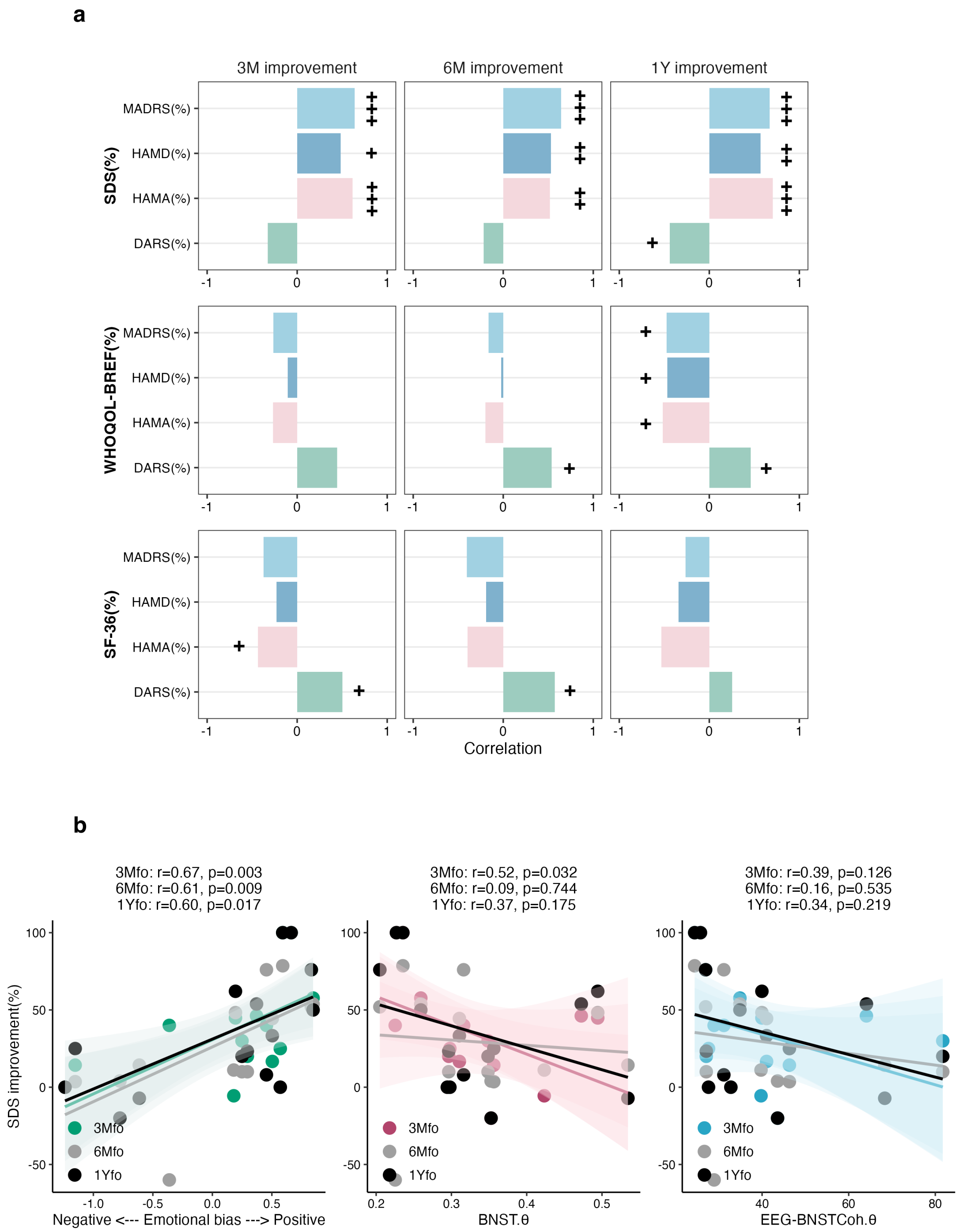

Supplementary Fig. 4: Predictors of quality-of-life and disability. a. Bar plot representing correlation (Spearman’s or Pearson’s r) between depression (HAMD, MADRS), anxiety (HAMA) and anhedonia (DARS) improvements and quality-of-life and disability outcomes at 3, 6 months and 1 year. $^{+}$ FDR adjusted

$p < 0.05$, ++ FDR adjusted $p < 0.01$, +++ FDR adjusted $p < 0.001$ for Spearman's correlation (two-sided). Source data are provided as a Source Data file. **b.** Linear regression prediction of 3-month (n = 17), 6-month (n = 17,) and 1-year (n = 15) SDS improvements by negative emotional bias, BNST theta power and theta coherence between frontal EEG and BNST in the eyes-closed dataset. Regression line shows the fitted mean and shading represents 95% CI (two-sided). Annotations report R^2 and P value for linear regression (two-sided). Abbreviations: HAMD, Hamilton Depression Scale; HAMA, Hamilton Anxiety Scale; MADRS, Montgomery-Asberg Depression Rating Scale; DARS, Dimensional Anhedonia Rating Scale; 3M, 3 months; 6M, 6 months; 12M, 12 months; fo, follow-up; Coh, coherence; SDS, Sheehan Disability Scale; WHOQOL-BREF, World Health Organization Quality of Life-BREF; SF-36, 36-Item Short Form Survey Instrument.

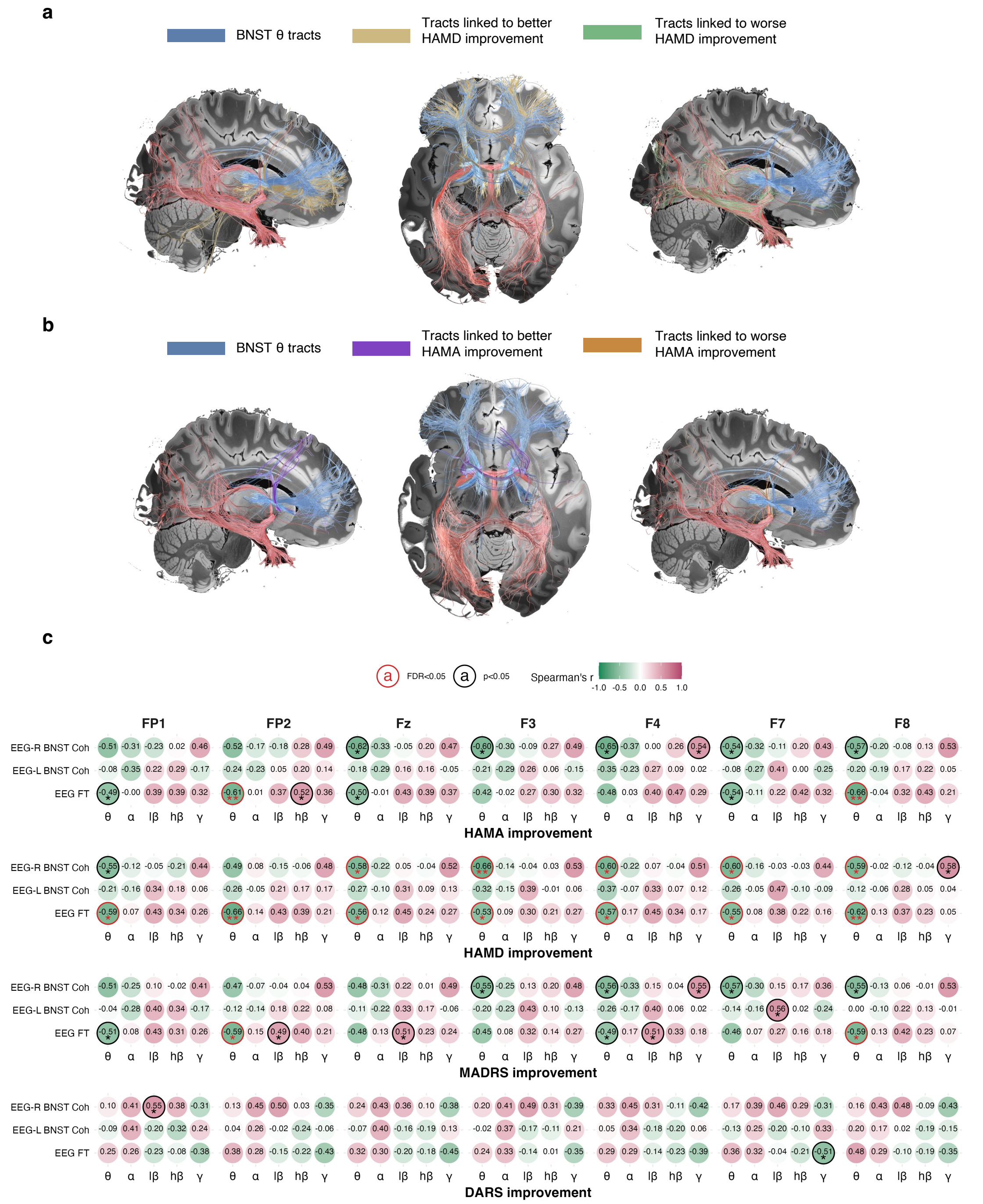

Supplementary Fig. 5: Hemispheric lateralization of theta-band biomarkers. a. Fiber tracts (blue) related to lower BNST theta power (predicting better clinical outcomes) overlaid with fiber tracts related to better (yellow) and worse (green) 3-month HAMD improvement. **b.** Fiber tracts (blue) related to lower BNST theta power overlaid with fiber tracts related to better

(purple) and worse (yellow) 3-month HAMA improvement. **c.** Correlational plots between spectral power, EEG-Left BNST and EEG-Right BNST coherence from each EEG channel, and improvements in depression (HAMD, MADRS), anxiety (HAMA), and anhedonia (DARS) at 3 months. Black circles indicate unadjusted $p < 0.05$ and red circles indicate FDR-adjusted $p < 0.05$ for Spearman's correlation (two-sided). Source data are provided as a Source Data file. Abbreviations: HAMD, Hamilton Depression Scale; HAMA, Hamilton Anxiety Scale; MADRS, Montgomery-Asberg Depression Rating Scale; DARS, Dimensional Anhedonia Rating Scale; L BNST, left bed nucleus of the stria terminalis; R BNST, right bed nucleus of the stria terminalis; FT, Fourier Transform; Coh, coherence.
