# Supplementary material for: Prefrontal–bed nucleus of the stria terminalis physiological and neuropsychological biomarkers predict therapeutic outcomes in depression
Source: Nat Commun. 2025 Nov 18;16:10034. doi: 10.1038/s41467-025-65179-z (PMC12627847; doi:10.1038/s41467-025-65179-z)
Supplement: Supplementary file 2 — Reporting Summary [file 41467_2025_65179_MOESM2_ESM.pdf]

nature portfolio

Corresponding author(s): Valerie Voon

Last updated by author(s): Aug 27, 2025

Reporting Summary

Nature Portfolio wishes to improve the reproducibility of the work that we publish. This form provides structure for consistency and transparency in reporting. For further information on Nature Portfolio policies, see our Editorial Policies and the Editorial Policy Checklist.

Statistics

For all statistical analyses, confirm that the following items are present in the figure legend, table legend, main text, or Methods section.

n/a	Confirmed	
☐	☒	The exact sample size (*n*) for each experimental group/condition, given as a discrete number and unit of measurement
☐	☒	A statement on whether measurements were taken from distinct samples or whether the same sample was measured repeatedly
☐	☒	The statistical test(s) used AND whether they are one- or two-sided<br>*Only common tests should be described solely by name; describe more complex techniques in the Methods section.*
☒	☐	A description of all covariates tested
☐	☒	A description of any assumptions or corrections, such as tests of normality and adjustment for multiple comparisons
☐	☒	A full description of the statistical parameters including central tendency (e.g. means) or other basic estimates (e.g. regression coefficient) AND variation (e.g. standard deviation) or associated estimates of uncertainty (e.g. confidence intervals)
☐	☒	For null hypothesis testing, the test statistic (e.g. *F*, *t*, *r*) with confidence intervals, effect sizes, degrees of freedom and *P* value noted<br>*Give P values as exact values whenever suitable.*
☒	☐	For Bayesian analysis, information on the choice of priors and Markov chain Monte Carlo settings
☒	☐	For hierarchical and complex designs, identification of the appropriate level for tests and full reporting of outcomes
☐	☒	Estimates of effect sizes (e.g. Cohen's *d*, Pearson's *r*), indicating how they were calculated

Our web collection on statistics for biologists contains articles on many of the points above.

Software and code

Policy information about availability of computer code

Data collection	Peri-operative Signals were recorded through BrainVision recorder 1.2. Wireless data was obtained through the interface SceneRay LFP_1.0.0.17257.
Data analysis	For data analysis, we used Lead-DBS v2 (http://www.lead-dbs.org) for lead reconstruction, VTA and connectivity estimation; EEGLAB toolbox v2022.1 for physiological data preprocessing; and MVGC multivariate Granger causality toolbox for Granger prediction estimation. Data analyses were performed using SPSS 20.0, Python 3.11, and Matlab R2023a. The figures were generated using R version 4.3.1.The code used to produce the results in this paper is available at https://github.com/Fallenworm/BNSTDBS.

For manuscripts utilizing custom algorithms or software that are central to the research but not yet described in published literature, software must be made available to editors and reviewers. We strongly encourage code deposition in a community repository (e.g. GitHub). See the Nature Portfolio guidelines for submitting code & software for further information.

Data

Policy information about availability of data

All manuscripts must include a data availability statement. This statement should provide the following information, where applicable:

- Accession codes, unique identifiers, or web links for publicly available datasets
- A description of any restrictions on data availability
- For clinical datasets or third party data, please ensure that the statement adheres to our policy

Provide your data availability statement here.

Research involving human participants, their data, or biological material

Policy information about studies with human participants or human data. See also policy information about sex, gender (identity/presentation), and sexual orientation and race, ethnicity and racism.

Reporting on sex and gender	We requested participants' age, biological sex, occupation, and basic demographic details. The sex category comprises options of male, female, and other. However, as these details are not pertinent to the focus of this study, they are not elaborated upon in the description but are solely included in the patient demographics section. Gender and ethics were self-reported by patients.
Reporting on race, ethnicity, or other socially relevant groupings	Race, ethnicity, or socially relevant groupings are not relevant to the present working. They are not reported.
Population characteristics	See Extended data Table 1 for details
Recruitment	We recruited refractory MDD patients scheduled to undergo DBS surgery at the Ruijin Hospital, Shanghai Jiaotong University School of Medicine for the study
Ethics oversight	The protocol was approved by the Ruijin Hospital Ethics Committee Shanghai Jiaotong University School of Medicine.

Note that full information on the approval of the study protocol must also be provided in the manuscript.

Field-specific reporting

Please select the one below that is the best fit for your research. If you are not sure, read the appropriate sections before making your selection.

- [x] Life sciences
- [ ] Behavioural & social sciences
- [ ] Ecological, evolutionary & environmental sciences

For a reference copy of the document with all sections, see nature.com/documents/nr-reporting-summary-flat.pdf

Life sciences study design

All studies must disclose on these points even when the disclosure is negative.

Sample size	The sample size was based on the assumption that a mean reduction of 9 points on HAMD score with a standard deviation of 8 when compared active DBS with sham DBS7. Consequently, it was estimated that 17 patients in the crossover phase would be sufficient to evaluate the efficacy of the trial with a type I error rate of 0.05 and a type II error rate of 0.9.
Data exclusions	A total of 524 patients were screened for eligibility, of which 26 were included in the study. During the open-label phase, three patients withdrew from the study at months 3, 9, and 10 of optimization. Additionally, five patients were not randomized in the cross-over trial. Consequently, 18 patients entered the crossover phase, with eight randomized to active-sham and 10 to sham-active DBS.
Replication	This study is based on a clinical trial, which by nature cannot be independently replicated. However, to ensure robustness, all analyses were independently replicated twice, yielding consistent results.
Randomization	We employed an envelope procedure for randomization, wherein each randomly assigned group was enclosed within a sealed, opaque envelope. An independent researcher sequentially opened these envelopes and conducted the randomization by switching DBS on or off accordingly.
Blinding	Throughout the trial, patients, two assessors (YW, LD, psychiatrists), and other relevant researchers were kept bAlinded to the stimulation condition. This double-blinding protocol remained in effect until the completion of the crossover phase by the last patient.

Reporting for specific materials, systems and methods

We require information from authors about some types of materials, experimental systems and methods used in many studies. Here, indicate whether each material, system or method listed is relevant to your study. If you are not sure if a list item applies to your research, read the appropriate section before selecting a response.

Materials & experimental systems

n/a	Involved in the study
☒	☐ Antibodies
☒	☐ Eukaryotic cell lines
☒	☐ Palaeontology and archaeology
☒	☐ Animals and other organisms
☐	☒ Clinical data
☒	☐ Dual use research of concern
☒	☐ Plants

Methods

n/a	Involved in the study
☒	☐ ChIP-seq
☒	☐ Flow cytometry
☐	☒ MRI-based neuroimaging

Clinical data

Policy information about clinical studies

All manuscripts should comply with the ICMJE guidelines for publication of clinical research and a completed CONSORT checklist must be included with all submissions.

Clinical trial registration: NCT04530942

Study protocol: https://classic.clinicaltrials.gov/ct2/show/NCT04530942?term=NCT04530942&cond=depression&draw=2&rank=1

Data collection: We recruited refractory MDD patients scheduled to undergo DBS surgery at the Ruijin Hospital, Shanghai Jiaotong University School of Medicine for the study from March 29th, 2021, to July 28th, 2023.. All data collection were completed during this period.

Outcomes:

Primary Outcome Measures ：
Effect size of active compared to sham stimulation score before and after the sham and treatment periods. The score of HAMD-17 ranges from 0 to 50. Higher HAMD-17 score indicates more severe depression.
[Time Frame: Baseline (preoperative),two weeks, one month, 3 months, 6 months, 9 months,10 months, 11 months, 12 months, 18months]

Secondary Outcome Measures ：
changes in the Montgomery-Asberg Depression Rating Scale(MADRS)
Effect size of active compared to sham stimulation score before and after the sham and treatment periods. The score of the scale ranges from 0 to 60. Higher MADRS score indicates more severe depression.
[Time Frame: Baseline (preoperative),two weeks, one month, 3 months, 6 months, 9 months,10 months, 11 months, 12 months, 18months]
changes in the Quick Inventory of Depression Scale(QIDS-SR16)
Effect size of active compared to sham stimulation score before and after the sham and treatment periods. The score of the scale ranges from 0 to 42. Higher QIDS-SR16 score indicates more severe depression.
[Time Frame: Baseline (preoperative),two weeks, one month, 3 months, 6 months, 9 months,10 months, 11 months, 12 months, 18months]
changes in the Depression and Somatic Symptoms Scale(DSSS)
Effect size of active compared to sham stimulation score before and after the sham and treatment periods. The score of the scale ranges from 0 to 66. Higher DSSS score indicates more severe depression and anxiety.
[Time Frame: Baseline (preoperative),two weeks, one month, 3 months, 6 months, 9 months,10 months, 11 months, 12 months, 18months]
changes in Hamilton Anxiety Scales(HAMA)
Clinician administered assessment.The score of the scale ranges from 0 to 56. The higher scores means more severe anxiety.
[Time Frame: Baseline (preoperative),two weeks, one month, 3 months, 6 months, 9 months,10 months, 11 months, 12 months, 18months]
changes in World Health Organization Quality of Life-BREF(WHO-BREF)
The World Health Organization Quality of Life - BREF (WHOQOL-BREF) is a self report questionnaire which assesses 4 domains of quality of life (QOL): physical health, psychological health, social relationships, and environment. It contains 26 items which is a 5 points scale. The higher score means better quality of life.
[Time Frame: Baseline (preoperative),two weeks, one month, 3 months, 6 months, 9 months,10 months, 11 months, 12 months, 18months]
changes in the MOS item short from health survey (SF-36)
SF-36 is a set of generic, coherent, and easily administered quality-of-life measures. These measures rely upon patient self-reporting and are now widely utilized by managed care organizations and by Medicare for routine monitoring and assessment of care outcomes in adult patients. The higher score means better quality of life.
[Time Frame: Baseline (preoperative),two weeks, one month, 3 months, 6 months, 9 months,10 months, 11 months, 12 months, 18months]
changes in Quality of Life Enjoyment and Satisfaction Questionnaire - Short Form
The scoring of the Q-LES-Q-SF involves summing only the first 14 items to yield a raw total score. The last two items are not included in the total score but are stand-alone items. The raw total score ranges from 14 to 70. The higher score means better quality of life.
[Time Frame: Baseline (preoperative),two weeks, one month, 3 months, 6 months, 9 months,10 months, 11 months, 12 months, 18months]
changes in Sheehan Disability Scale

Self-rating scale. The SDS is a composite of three self-rated items designed to measure the extent to which three major domains in the patient's life are functionally impaired by psychiatric or medical symptoms. The SDS assesses functional impairment in three major life domains: work, social life/leisure activities, and family life/home responsibilities. The higher scores mean more severity of disability.
[Time Frame: Baseline (preoperative),two weeks, one month, 3 months, 6 months, 9 months,10 months, 11 months, 12 months, 18months]
changes in Neuropsychological measures(Scores of CANTAB tasks)
Neuropsychological measures contains six tasks which are Stop Signal Task, Spatial Working Memory, Paired Associated Learning, Stocking of Cambridge, Intra Extra dimensional Set Shifting, Rapid visual processing task, emotional recognition task, emotional bias task
[Time Frame: Baseline (preoperative),10 months, 11 months, 18months]

Plants

Seed stocks — *Report on the source of all seed stocks or other plant material used. If applicable, state the seed stock centre and catalogue number. If plant specimens were collected from the field, describe the collection location, date and sampling procedures.*

Novel plant genotypes — *Describe the methods by which all novel plant genotypes were produced. This includes those generated by transgenic approaches, gene editing, chemical/radiation-based mutagenesis and hybridization. For transgenic lines, describe the transformation method, the number of independent lines analyzed and the generation upon which experiments were performed. For gene-edited lines, describe the editor used, the endogenous sequence targeted for editing, the targeting guide RNA sequence (if applicable) and how the editor was applied.*

Authentication — *Describe any authentication procedures for each seed stock used or novel genotype generated. Describe any experiments used to assess the effect of a mutation and, where applicable, how potential secondary effects (e.g. second site T-DNA insertions, mosiacism, off-target gene editing) were examined.*

Magnetic resonance imaging

Experimental design

Design type — Resting state

Design specifications — 1 block for resting state imaging.

Behavioral performance measures — Resting-state imaging did not involve the assessment of any behavioral measures.

Acquisition

Imaging type(s) — Structural

Field strength — 3T

Sequence & imaging parameters — High resolution T1-weighted (T1w) anatomical images (TFE, TE/TR/TI = 3.4/7.1/938.2ms, FA = 7°, FOV = 240*240mm, TA=3'14'') and T2-weighted (T2w) images (TSE, TE/TR= 106/74000ms, FA = 90°, FOV = 240*240mm, TA=5'36'') were acquired preoperatively, and CT (thickness=3.75mm) was acquired postoperatively

Area of acquisition — Whole brain scan

Diffusion MRI ☐ Used ☒ Not used

Preprocessing

Preprocessing software — Automatic brain reconstruction and DBS electrode localization utilized Lead-DBS v2 (http://www.lead-dbs.org).

Normalization — Postoperative CT images were first linearly co-registered to preoperative MRI and normalized into ICBM 2009b NLIN asymmetric space using the SyN approach implemented in Advanced Normalization Tools.

Normalization template — ICBM 2009b NLIN asymmetric space

Noise and artifact removal — The procedure didn't involve noise and artifact removal.

Volume censoring — The procedure didn't involve volume censoring.

Statistical modeling & inference

Model type and settings — Spearman rank correlation analysis was performed to examine the relationship between structural connectivity and the identified physiology in the patient cohort.

Effect(s) tested — Spearman rank correlation analysis

Specify type of analysis: ☐ Whole brain ☒ ROI-based ☐ Both

Anatomical location(s)

DBS electrodes were then localized using Lead-DBS and warped into the Montreal Neurological Institute (MNI) space using the PaCER algorithm after visual review and refinement of the co-registrations and normalizations.

Statistic type for inference

(See Eklund et al. 2016)

Specifically, the E-Field was estimated using a finite element method on a four-compartment mesh describing local grey and white matter, as well as electrode contact and insulating material. Subsequently, voxel-wised normative structural connectivity seeding from bilateral E-fields were estimated using normative data sets retrieved from the Human Connectome Project at Massa-chusetts General Hospital (32 subjects, multi-shell diffusion-weighted imaging data). E-field values were used as weights to construct connectivity profiles. For each patient, fibers passing through a non-zero voxel of the E-field were extracted from the normative connectome and mapped onto a standardized voxelized volume with 2mm resolution. Each fiber received the weight of the maximal E-field magnitude of its passage and fiber densities were weighted by these values. Spearman rank correlation analysis was then performed to examine the relationship between structural connectivity and the identified physiology in the patient cohort. The uncorrected significant fiber tracts were integrated to construct the correlated map (Rmap).

Correction

uncorrrected

Models & analysis

n/a	Involved in the study
☒	☐ Functional and/or effective connectivity
☒	☐ Graph analysis
☒	☐ Multivariate modeling or predictive analysis
